# Remembered Meal Satisfaction, Satiety, and Later Snack Food Intake: A Laboratory Study

**DOI:** 10.3390/nu10121883

**Published:** 2018-12-03

**Authors:** Victoria Whitelock, Eric Robinson

**Affiliations:** 1 Department of Psychological Sciences, University of Liverpool, Eleanor Rathbone Building, Bedford Street South, Liverpool L69 7ZA, UK; 2 Cancer Intelligence, Cancer Research UK, Angel Building, 407 St John Street, London EC1V 4AD, UK

**Keywords:** memory, remembered satisfaction, satiety, eating behavior

## Abstract

It is well established that the satiety providing effects of food can influence meal size and a disparate area of research suggests that memory regarding recent eating informs food intake. Here we examined whether remembered meal satisfaction (encompassing memory for meal liking and satiety) can be manipulated in the laboratory and whether this influences later food intake. Participants (*N* = 128, body mass index mean = 23.46kg/m^2^, standard deviation = 4.70) consumed a fixed lunch and then rehearsed the satisfying or dissatisfying aspects of the meal, or a neutral experience (control), in order to manipulate memory for meal satisfaction. Three hours later participants completed a bogus taste-test to measure food intake and meal memory measures. There was no evidence that memory for general satisfaction with the meal was affected by the rehearsal condition. However, in the dissatisfying rehearsal condition, participants remembered being less satisfied with the satiety-providing effects of the lunch meal than in the satisfying and neutral rehearsal conditions. Snack food consumption did not differ across conditions and there was a small negative correlation between how satiating participants remembered their earlier meal to be and later snack food intake (*r* = −0.16, *p* = 0.07). The present study did not produce evidence that memory relating to meal satiety affects later food intake but further research is warranted.

## 1. Introduction

Satiety has long been known to be an important concept in understanding the eating behavior of humans [1]. Of particular note, is more recent evidence which suggests that expectations concerning the satiety providing effects of a meal determine how much people are likely to serve themselves and eat [2,3]. Meal satisfaction is a less well studied concept in eating behaviour but is likely to be, in part, related to and determined by the satiety providing effects of a meal. In observational studies conducted in the laboratory both satisfaction with the sensory experience during meal consumption (e.g., liking of the taste experience) and satisfaction with the post-meal consequences of consuming that meal (e.g., how satiating the meal was) have been found to determine meal satisfaction [4]. These findings suggest that satisfaction with both the taste and satiety providing effects of a meal are distinct but, nonetheless, strongly contribute to the overall satisfaction with a meal. Meal satisfaction has been identified as a potentially important driver of human eating behaviour [5]. Moreover, given that when making food choices we are reliant on our memories of past eating experiences [6,7], meals that are remembered as being satisfying, as opposed to dissatisfying will be more likely to be chosen again. However, the role that memory for meal satisfaction has on intake of other foods and the regulation of food intake more generally has received little attention. In the present study, we aimed to examine whether manipulating memory for meal satisfaction (including meal satiety) affects later intake of other foods. 

We reason that memory for how satisfying a recent meal has been may play a role in determining later intake of other foods because a number of studies suggest that memory for recent eating affects appetite regulation. For example, humans with damage to brain areas relating to memory have been found to eat multiple consecutive meals in the absence of any memory for having just eaten a meal [8,9]. In healthy, neurologically intact humans, eating a meal while distracted (e.g., watching TV, playing a computer game) has been shown to lead to poorer memory encoding for the meal compared to participants who did not eat while distracted. Participants with poorer memory for the earlier meal then ate more snacks later the same day [10,11,12]. In contrast to impaired memory, cueing memory for a meal consumed a few hours earlier has been found to reduce snack intake [13]. In further support, participants who paid focused attention to a lunchtime meal had a more vivid memory for that meal and ate fewer snacks 2–3 h later compared to controls [14]. However, recent work in our laboratory failed to find an effect of focused attention on the memory for a lunchtime meal or on snack intake 3 h later in two studies [15]. Although this area of research generally suggests a role of memory for recent eating in appetite regulation, it is not clear which aspects of memory for recent eating inform subsequent food intake. 

Memory for the satiety providing effects of a recent meal has been proposed to be an important underlying process which explains how memory guides food intake [13,16], presumably in part because remembering a lack of satisfaction with the satiety providing effects of a meal may increase later food intake. However, no research we are aware of has specifically examined the role that memory for recent meal satisfaction (and/or memory for the satiety providing effects of the meal) have in shaping food intake. In the current study we attempted to experimentally manipulate remembered meal satisfaction by instructing participants to rehearse either the satisfying or dissatisfying aspects of a meal after eating it; a method that has been successfully used to manipulate remembered enjoyment of a meal [17]. We then observed the effect this had on snack food intake three hours later. We hypothesised that rehearsing the satisfying (dissatisfying) aspects of a lunchtime meal would increase (decrease) remembered satisfaction with the meal and reduce (increase) snack intake compared to a control condition.

## 2. Materials and Methods 

### 2.1. Design and Sample

The study design and analysis strategy were pre-registered on the Open Science Framework, and can be found here [18]. Participants were told that they were taking part in two separate studies which aimed to examine the effect of personality on perceptions of savoury food (“study 1”, lunchtime session) and sweet food (“study 2”, afternoon session). Using a between-subjects design, participants consumed a fixed cold pasta meal during the lunchtime session and subsequently rehearsed the satisfying or dissatisfying aspects of the meal, or rehearsed a neutral experience in a control condition (their journey to the university campus). During the afternoon session three hours later participants completed a bogus taste-test (measure of later food intake) and measures of memory for satisfaction with the earlier lunch. We opted for three hours between the two sessions as based on previous laboratory research this time period would allow for memory effects on later food intake to be observed [14]. Participants gave written informed consent to take part in the study. The study was conducted following the rules of the Declaration of Helsinki and was approved by the University of Liverpool Research Ethics Committee (project number: IPHS-1617-LB-280-Generic RETH000955).

### 2.2. Participants

Participants were men and women aged between 18–60 years old, with fluent English, not taking medication that affects appetite, and had no known history of food allergies or disordered eating. The effect of manipulating remembered satisfaction on later food intake has not been investigated before. Therefore, we planned to recruit 40 participants per condition (*N* = 120) which would power the study to detect what would be considered to be close to a medium-sized statistical effect of experimental conditions (*f* = 0.29, 80% power, *p* < 0.05) on taste-test food intake (kilocalories; kcal). To account for having to exclude a small number of participants from analyses (e.g., extreme outliers on dependent variables) we planned to recruit up to 46 participants per cell (*N* = 138). 

### 2.3. Randomisation and Blinding

Randomisation to a condition was stratified by gender to ensure an equal gender distribution across conditions, and the allocation sequence was created using the Sealed Envelope Ltd, UK website (https://sealedenvelope.com/). The researchers running the study were blinded to the experimental condition participants were allocated to.

### 2.4. Lunchtime Meal

The lunchtime meal was a fixed 400 g portion of ASDA tomato and basil pasta salad (suitable for vegetarians, 600 kcal). This amount of pasta was chosen to be consistent with UK public health nutrition guidelines of 600 kcal per main meal [19,20] and it provided a meal size that was consistent with the amount of food participants from this population tend to eat in other studies in our laboratory [21]. Participants were also provided with a 200 mL glass of water. A fixed lunchtime meal was used to control for the volume of food eaten and is in line with previous studies [14,15].

### 2.5. Bogus Taste-Test

The bogus taste-test is a valid measure of food intake [22]. In this study, participants were provided with three bowls of biscuits broken up into pieces to reduce monitoring (3 × 50 g each of Maryland chocolate chip cookies, Cadbury’s chocolate fingers, and McVities digestives; ~742 kcal). Biscuit consumption was calculated by subtracting the post-taste-test weight from the pre-taste-test weight, converted to kilocalories and summed across the three biscuit types to produce our main dependent variable (total snack intake in kcal).

### 2.6. Memory Manipulation

After eating lunch, participants were asked to spend 6 min writing about either the satisfying or dissatisfying aspects of the meal, or a neutral event. In the satisfying rehearsal condition, the instructions were as follows: ‘Please write down your thoughts on what you found satisfying about the meal you just ate. Consider the following things: what did you like about the taste/flavour, appearance, smell, texture, and how the meal made you feel?’ The same instructions were given to the dissatisfying rehearsal condition, except that they were asked to rehearse what they found dissatisfying and what they disliked about the meal. Participants in the neutral rehearsal condition were asked to describe their journey to campus that day and to provide as much detail as possible. We reasoned that the two experimental manipulations would serve to increase (satisfying rehearsal condition) and decrease (dissatisfying rehearsal condition) overall memory for meal satisfaction by changing memory for satisfaction with the taste and satiety providing properties of the meal. 

### 2.7. Remembered Satisfaction

To measure remembered satisfaction with the lunchtime meal, participants responded to seven items using 100-point visual analogue response scales (anchors: not at all, extremely). To measure memory for overall meal satisfaction: ‘Overall, how satisfying did you find the lunchtime meal?’, ‘Overall, how dissatisfying did you find the lunchtime meal?’ and ‘I liked the lunchtime meal’. To measure memory for meal satiety: ‘How satisfied were you with how filling the lunchtime meal was?’, ‘How dissatisfied were you with how filling the lunchtime meal was?’ To measure memory for the taste of the meal: ‘How satisfied were you with the taste of the lunchtime meal?’ ‘How dissatisfied were you with the taste of the lunchtime meal?’ We hypothesised that these items would load onto a single factor (representing meal satisfaction), but planned to formally test for evidence of this by using factor analysis. 

### 2.8. Measures

In order to characterise the participant sample, measures of age and dietary habits were included (Three-Factor Eating Questionnaire, TFEQ: measuring cognitive restraint, uncontrolled eating, and emotional eating) [23]. We also included a 24-item personality questionnaire to bolster the cover story [24].

### 2.9. Procedure

After being screened for eligibility over email (to check self-reported age, English fluency, medications, and disordered eating), participants were invited to attend the lunchtime session (“study 1”) and ensure that they were available to come back 3 h later to complete the afternoon session (“study 2”). Participants were asked to eat their usual breakfast on the day of participation and abstain from eating 2 h prior to the lunchtime session. After providing consent and being asked not to use their mobile phones away for the duration of the study, participants completed a brief medical history questionnaire to ensure they did not have any food-related allergies and a brief (non-validated) English language test to ensure they were fluent in English. The medical history questionnaire asked participants about previous allergic reactions to a range of foods and medication use. Participants then completed the first 12 items from the personality questionnaire (in line with the cover story) and a set of appetite and mood ratings on 100-point visual analogue scales (anchors: ‘not at all’ to ‘extremely’) to measure hunger, fullness (e.g., ‘how hungry do you feel right now?’) and various mood dimensions. The mood questions acted as a further distraction from the real aims of the study. Next, the researcher brought in the lunch. Participants were told that they had 10 min to eat all of the meal. After the lunch, participants were asked to complete the 6 min rehearsal task (based on condition allocation), followed by the appetite and mood ratings once again. At the end of this session, participants were asked to not consume any calorie-containing food or drink between the two studies.

When returning for the second session (“study 2”) three hours from the start of their first session, participants were asked to sign an additional consent form to bolster the cover story. Participants then completed the same appetite and mood rating scales as in session 1 in addition to twelve further items from the personality questionnaire. After this, participants completed the taste-test. Participants were asked to make a series of pen and paper taste-ratings about the biscuits on 100-point visual analogue scales (anchors: not at all, extremely), to bolster the taste-test cover story (e.g., which cookie is crunchiest, sweetest, etc.) and were left alone for ten minutes to do this. Participants were reminded not to use their mobile phone during the taste-test. Participants then completed the same appetite and mood ratings for a final time before completing the remembered satisfaction questions. Participants then provided demographic information, completed the TFEQ, and wrote down the last time they ate during the study day (to check compliance with instructions not to eat between study sessions). Awareness of the study aims was assessed using a funnelled debrief procedure where participants were asked to write down (1) ‘what do you think the overall aim of the research was?’ and (2) ‘based on what you did in study 1, how do you think we expected this to affect your behaviour in study 2?’ At the end of the second session participants were weighed and measured using electronic body weight scales and a stadiometer in order to calculate BMI (body mass index).

### 2.10. Analysis Strategy

SPSS (version 24) (Armonk, NY, USA) was used for all statistical analyses [25]. Five out of the seven questions measuring remembered satisfaction were normally distributed; the two questions measuring remembered satisfaction regarding how filling the lunchtime meal was were slightly skewed. Contrary to our expectations, the seven questions measuring remembered satisfaction for the meal were not all strongly correlated (Pearson’s correlation results are reported in Table S1 in the Supplementary Materials). Principal components analysis ^(a)^ with varimax rotation yielded a 2-factor solution with eigenvalues above 1 [26], and a “levelling off” in the scree plot confirmed that this was appropriate [27]. Factor one explained 64% of the variance and factor two explained 23% of the variance. The two questions assessing memory for meal satiety loaded onto one factor and the other five questions loaded onto the other factor. Therefore, we averaged items from these two individual factors to create distinct measures of (1) remembered satisfaction with meal satiety and (2) remembered general satisfaction with the lunchtime meal, with the latter encompassing memory for general liking and enjoyment of the meal. The effect of a rehearsal condition (between-subject factor) on hunger across study time-points (repeated measures factor: pre-lunch, post-lunch, pre-ad-libitum taste-test, post-ad-libitum taste-test) was examined using mixed analysis of variance (ANOVA) ^(b)^. One-way ANOVAs were used to examine the effect of the rehearsal condition on total snack food intake and the two measures of meal memory. Least Significant Difference (LSD) post hoc comparisons were used to follow up significant effects. Bootstrapped confidence intervals (CIs) (1000 samples) are provided for the post hoc comparisons for remembered satisfaction with meal satiety (as the questions that constitute this measure were slightly skewed). The dataset for this study is available on the UK Data Service (http://dx.doi.org/10.5255/UKDA-SN-853370).

(a) The use of principle components analysis was not explicitly stated in the pre-registered protocol in error.
(b) One-way ANOVAs were planned in the pre-registered protocol in error, as a mixed ANOVA accounting for repeated measurement is more appropriate.

## 3. Results

The datasets generated and analysed during the current study are available from the UK Data Service ReShare repository (http://dx.doi.org/10.5255/UKDA-SN-853370).

### 3.1. Sample

Participants were members of staff and students at the University of Liverpool or members of the community. During data collection, we noted that a minority of participants did not eat all of the fixed lunch, which was a pre-specified criterion for exclusion from analyses. Therefore, we recruited slightly more participants than originally planned to ensure we reached our minimum sample size. Out of the 146 participants that completed the study, in line with our pre-registered exclusion criteria, eighteen were excluded from the main analyses for not following the study instructions: *n* = 12 because they ate less than 90% of the lunch (we used 90% as a cut off to account for any food waste from evaporation and residual pasta sauce left on the plate), *n* = 1 because they used their mobile phone during the taste-test, *n* = 3 because they ate in between the study sessions, and *n* = 2 for providing a very minimal response on the rehearsal task. The final analysed sample was *N* = 128 (71.1% female; neutral rehearsal = 44; satisfying rehearsal = 43; dissatisfying rehearsal = 41). Mean BMI on the day of testing was 23.46 (SD = 4.70). See Table 1 for sample characteristics split by condition.

### 3.2. Memory Measures 

There was a non-significant effect of the rehearsal condition on memory for general satisfaction with the lunchtime meal, *F*(2,125) = 1.86, *p* = 0.16, and *η^2^* = 0.03. However, there was a significant effect of rehearsal condition on memory for satisfaction with meal satiety, *F*(2,125) = 5.50, *p* = 0.005, and *η^2^* =0.08. Those experiencing the dissatisfying rehearsal condition remembered being significantly less satisfied with how satiating the lunchtime meal was compared to participants in both the neutral rehearsal (*p* = 0.01, bootstrapped 95% (CI) −22.47 to −1.86) and satisfying rehearsal conditions (*p* = 0.002, bootstrapped 95% CI −25.02 to −5.50). The participants in the satisfying and neutral rehearsal conditions did not significantly differ on memory for satisfaction with meal satiety (*p* = 0.57, bootstrapped 95% CI −5.98 to 11.46), see Table 2. Thus, the experimental manipulation did not affect how satisfying the meal was remembered to be in general but did affect memory for the satiety providing effects of the meal.

### 3.3. Hunger

There was a non-significant effect of condition on hunger, *F*(2,125) = 1.67, *p* = 0.19, and *η^2^* = 0.03. There was a significant effect of study time-point on hunger, *F*(2.61,325.80) = 305.10, *p* < 0.001, and *η^2^* = 0.71 (Greenhouse–Geisser corrected due to violation of the sphericity assumption), such that hunger was significantly lower post-lunch compared to pre-lunch, *F*(1,125) = 738.86, *p* < 0.001, and *η^2^* = 0.86, greater at pre-ad-libitum taste-test compared to post-lunch, *F*(1,125) = 221.72, *p* < 0.001, *η^2^* = 0.64, and lower at post-ad-libitum taste-test compared to pre ad-libitum taste-test, *F*(1,125) = 129.70, *p* < 0.001, and *η^2^* =0.51. The interaction between condition and time-point on hunger was non-significant, *F*(5.21,325.80) = 0.21, *p* = 0.96, and *η^2^* < 0.01, see Table 2.

### 3.4. Ad libitum Snack Intake

**Figure 1:**
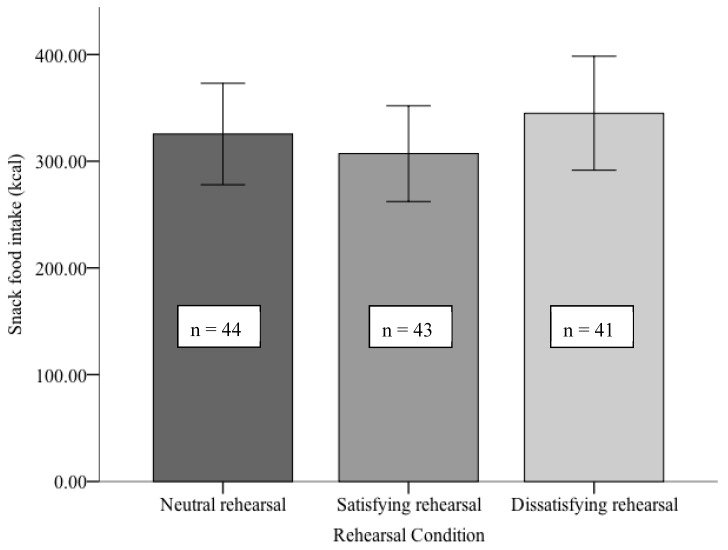
Snack food intake as a function of rehearsal condition (error bars represent 95% confidence intervals).

Contrary to the hypothesis, there was no significant effect of rehearsal condition on ad libitum snack intake, *F*(2,125) = 0.61, *p* = 0.55, and *η^2^* = 0.01, see Figure 1.

### 3.5. Sensitivity Analyses

There were no outliers on total snack food intake, and no participants correctly guessed the exact aims of the study. However, three participants reported that they expected satisfaction with the lunchtime meal to influence later snack food intake. Excluding these participants had no effect on the results for snack intake. Including BMI, dietary restraint, and uncontrolled eating as covariates also did not affect the results for snack food intake. See Supplementary Materials for full statistical information.

### 3.6. Exploratory Analyses

Some participants did not complete the rehearsal task as fully intended (e.g., some participants also mentioned satisfying aspects of the meal when asked to write about the dissatisfying aspects and vice versa). Therefore, we examined the effect of limiting our main analyses to participants that only wrote about satisfying aspects of the meal in the satisfying rehearsal condition and likewise for the dissatisfying rehearsal condition. One researcher coded participants who described satisfying (dissatisfying) aspects of the meal when instructed to write about the dissatisfying (satisfying) aspects of the meal. A second researcher cross-checked the coded rehearsal task responses, and any disagreements were resolved by discussion. The results are reported in Table S2 in the Supplementary Material, although this did not affect the main result of condition on snack food intake. Further, in the full sample, we also examined whether either memory measure was associated with later snack food intake. Snack food intake did not significantly correlate with remembered general satisfaction; *r* = −0.11, *p* = 0.21 or remembered satisfaction with meal satiety; *r* = −0.16, *p* = 0.07.

## 4. Discussion

In the present study, we examined the effect of rehearsing the satisfying or dissatisfying aspects of a lunchtime meal on memory for that meal and later food intake. Results showed that rehearsal condition had no impact on a measure of memory for general satisfaction with the earlier meal, but did affect a measure of memory for satisfaction with the satiety providing effects of the meal; participants that rehearsed the dissatisfying aspects of the meal remembered feeling less satisfied with how satiating the meal was compared to participants who rehearsed the satisfying aspects of the meal and participants in a non-meal rehearsal control condition. However, there was no effect of rehearsal condition on later food intake.

Our results were not in line with our expectations as we had expected that rehearsal condition would affect memory for overall satisfaction of the meal. We attempted to measure overall satisfaction through individual self-report items on meal satisfaction in general, as well as satisfaction with the taste and satiety providing effects of the meal. However, when we examined this measure, results indicated a two-factor structure; one relating to memory for the satiety providing effects of the meal and the other encompassing sensory (taste) and more general satisfaction with the meal. Therefore, it appears that when asked to report on the overall satisfaction with the meal, participants were relying on the taste or sensory aspects of the meal, but not the satiety providing effects of the meal. Based on this, we conclude that our manipulation was not successful in manipulating memory for overall satisfaction. However, our manipulation did affect memory for meal satiety and this is the first study we are aware of to do this and examine the effect this has on later food intake. Although our manipulation caused participants to remember being relatively dissatisfied with how satiating their lunchtime meal was, this did not, in turn, affect how much they chose to eat three hours later. 

It is not clear why a manipulation that resulted in participants remembering being relatively dissatisfied with the amount of satiety that an earlier meal produced did not affect later food intake. Memory for recent eating has been proposed to inform appetite regulation because it provides information about the satiety providing effects of earlier meals [13,16]. Therefore, it is possible that this theoretical proposal is not accurate, is context dependent, or that our study methodology explains the non-significant effects observed on food intake. One explanation is that, although we based the experimental manipulation on previous work [17], the manipulation used did not produce a large enough change in memory for meal satiety in order to affect later food intake. We only observed a modest and non-significant association (*r* = −0.16, *p* = 0.07) between later food intake and how satisfied participants remembered being with the satiating effects of the meal, which may suggest that any effect of memory for earlier meal satiety on food intake would be minimal. That said, although our measure of memory for earlier meal satiety was sensitive to experimental manipulation we may not have measured this construct precisely enough. We made use of a self-devised two-item measure and it may be the case that this introduced measurement error. On this basis, we recommend that future research considers adopting other measures, preferably ones that have been validated. An additional consideration is that the size of the lunchtime meal served in the present study may have affected our findings. Some participants may have found the meal too large and this may have affected both memory and later food intake. Future research could consider providing meals that are tailored to individual energy requirements, rather than providing fixed meals as in the present and other studies [14,15,28].

### 4.1. Strengths and Limitations

Strengths of the present study are that we pre-registered our study methods and analysis plan and used a neutral rehearsal control condition, which allowed us to compare the independent effects of dissatisfying and satisfying rehearsal conditions. A further strength of the current study is that we measured several aspects of remembered meal satisfaction. Meal satisfaction appears to be multi-faceted and it is important for research to understand the individual components of memory for satisfaction. There are limitations to the present research. As noted, our measures of memory were self-devised for the purpose of the study and have therefore not been validated. The current findings should, therefore, be considered preliminary and in the context of the specific measures of memory for satisfaction used, and cannot be generalized to other aspects of memory for satisfaction that were not measured (see Implications and Future Research section for a discussion of other aspects of memory for meal satisfaction). Our manipulation was successful in manipulating memory for satisfaction with meal satiety but not memory for overall meal satisfaction. Therefore, although this permits us to draw conclusions about the effect of remembered satisfaction with meal satiety on later intake, it does not allow us to make any more general conclusions about memory for satisfaction. 

### 4.2. Implications and Future Research

The results of the present study suggest that remembered satisfaction with the satiety providing effects of a lunchtime meal is a construct that can be manipulated independently from other aspects of remembered meal satisfaction. Here, we did not find convincing evidence that either memory for general satisfaction with a meal (a measure of satisfaction encompassing ratings of meal taste and liking) or memory for the satiety providing effects of a meal were associated with food intake three hours later. However, this may be, in part, due to methodological considerations and further work in this area can now be more definitive. In this vein, future research is now warranted to investigate other aspects of remembered satisfaction that were not measured in this study (e.g., remembered satisfaction with the appearance of the food, volume of food, and meal performance relative to expectations), as this may allow for identification of whether specific aspects of remembered satisfaction with a meal are contributors to subsequent eating behaviours. Understanding such a process may benefit approaches that make use of remembered satisfaction as a way of reducing food consumption. Future research could also examine how initial liking of the food alters the effect of the memory manipulation and provide meals based on energy requirements instead of providing fixed-size meals as per the current study and previous studies [14,15,28].

## 5. Conclusions

Rehearsing the dissatisfying aspects of a lunchtime meal resulted in participants remembering being less satisfied with the satiety providing effects of an earlier meal. However, this did not influence later snack food intake. Therefore, the present study did not produce evidence that memory for meal satiety affects later food intake. Further research to understand the determinants of memory for meal satiety and satisfaction and their effects on appetite regulation would now be informative (Trial Registration: ClinicalTrials.gov, NCT03750019).

## Figures and Tables

**Table 1:** Sample characteristics as a function of rehearsal condition.

Characteristic	Neutral Rehearsal Mean (*SD*) *n* = 44	Satisfying Rehearsal Mean (*SD*) *n* = 43	Dissatisfying Rehearsal Mean (*SD*) *n* = 41
BMI (kg/m^2^)	23.55 (4.20)	23.73 (5.19)	23.09 (4.78)
Age (years)	25.59 (9.78)	23.79 (7.52)	23.46 (7.44)
TFEQ cognitive restraint	2.33 (0.62)	2.25 (0.54)	2.11 (0.54)
TFEQ uncontrolled eating	2.55 (0.57)	2.27 (0.56)	2.38 (0.47)
TFEQ emotional eating	2.34 (0.63)	2.17 (0.72)	2.22 (0.63)

Note. Range of possible scores: cognitive restraint, uncontrolled eating, emotional eating = 1–4. Higher scores indicate greater endorsement. TFEQ = Three-Factor Eating Questionnaire

**Table 2:** Remembered satisfaction and hunger as a function of rehearsal condition.

Variable	Neutral Rehearsal Mean (*SD*) *n* = 44	Satisfying Rehearsal Mean (*SD*) *n* = 43	Dissatisfying Rehearsal Mean (*SD*) *n* = 41
Remembered general satisfaction	53.35 (25.24)	52.73 (26.37)	43.72 (24.95)
Remembered satisfaction with meal satiety	80.31 (22.60)	83.02 (18.72)	67.84 (25.27)
Pre-lunch hunger	57.73 (18.71)	55.47 (19.66)	58.20 (23.17)
Post-lunch hunger	7.91 (11.19)	4.16 (6.03)	10.63 (12.44)
Pre-ad-libitum taste-test hunger	36.05 (21.25)	31.37 (21.29)	36.66 (23.92)
Post-ad-libitum taste-test hunger	14.36 (14.70)	12.44 (13.85)	17.51 (16.40)

Note. The response scale for all measures is 0–100, with higher scores indicating greater endorsement.

## Supplementary Materials

The following are available online at http://www.mdpi.com/2072-6643/10/12/1883/s1, Table S1: Correlation matrix for memory for satisfaction with the lunchtime meal questions, Table S2: Snack food intake as a function of recoded rehearsal condition.

## References

[1] Blundell J., Rogers P., Hill A., Solms J., Booth D., Pangbourne R., Raunhardt O. (1987). Evaluating the satiating power of foods: Implications for acceptance and consumption. Food Acceptance and Nutrition.

[2] Brunstrom J.M., Rogers P.J. (2009). How Many Calories Are on Our Plate? Expected Fullness, Not Liking, Determines Meal-size Selection. Obesity.

[3] Wilkinson L.L., Hinton E.C., Fay S.H., Ferriday D., Rogers P.J., Brunstrom J.M. (2012). Computer-based assessments of expected satiety predict behavioural measures of portion-size selection and food intake. Appetite.

[4] Vad Andersen B., Hyldig G. (2015). Food satisfaction: Integrating feelings before, during and after food intake. Food Qual. Prefer..

[5] Cardello A.V., Schutz H., Snow C., Lesher L. (2000). Predictors of food acceptance, consumption and satisfaction in specific eating situations. Food Qual. Prefer..

[6] Robinson E., Blissett J., Higgs S. (2011). Recall of Vegetable Eating Affects Future Predicted Enjoyment and Choice of Vegetables in British University Undergraduate Students. J. Am. Diet. Assoc..

[7] Robinson E. (2014). Relationships between expected, online and remembered enjoyment for food products. Appetite.

[8] Higgs S., Williamson A.C., Rotshtein P., Humphreys G.W. (2008). Sensory-Specific Satiety Is Intact in Amnesics Who Eat Multiple Meals. Psychol. Sci..

[9] Rozin P., Dow S., Moscovitch M., Rajaram S. (1998). What Causes Humans to Begin and End a Meal? A Role for Memory for What Has Been Eaten, as Evidenced by a Study of Multiple Meal Eating in Amnesic Patients. Psychol. Sci..

[10] Higgs S., Woodward M. (2009). Television watching during lunch increases afternoon snack intake of young women. Appetite.

[11] Mittal D., Stevenson R.J., Oaten M.J., Miller L.A. (2011). Snacking while watching TV impairs food recall and promotes food intake on a later TV free test meal. Appl. Cogn. Psychol..

[12] Oldham-Cooper R.E., Hardman C.A., Nicoll C.E., Rogers P.J., Brunstrom J.M. (2011). Playing a computer game during lunch affects fullness, memory for lunch, and later snack intake. Am. J. Clin. Nutr..

[13] Higgs S. (2002). Memory for recent eating and its influence on subsequent food intake. Appetite.

[14] Higgs S., Donohoe J.E. (2011). Focusing on food during lunch enhances lunch memory and decreases later snack intake. Appetite.

[15] Whitelock V., Higgs S., Brunstrom J.M., Halford J.C.G., Robinson E. (2018). No effect of focused attention whilst eating on later snack food intake: Two laboratory experiments. Appetite.

[16] Higgs S. (2015). Cognitive processing of food rewards. Appetite.

[17] Robinson E., Blissett J., Higgs S. (2012). Changing memory of food enjoyment to increase food liking, choice and intake. Br. J. Nutr..

[18] Memory for Meal Satisfaction and Subsequent Food Intake.

[19] A Quick Guide to the Government’s Healthy Eating Recommendations. https://assets.publishing.service.gov.uk/government/uploads/system/uploads/attachment_data/file/595133/A_quick_guide_to_govt_healthy_eating.pdf.

[20] The Scientific Principles for Developing Nutrient-Based Standards for Planning Nutritionally Balanced Menus. https://assets.publishing.service.gov.uk/government/uploads/system/uploads/attachment_data/file/648744/healthier_and_more_sustainable_nutrition_principles.pdf.

[21] Sheen F., Hardman C.A., Robinson E. (2018). Plate-clearing tendencies and portion size are independently associated with main meal food intake in women: A laboratory study. Appetite.

[22] Robinson E., Haynes A., Hardman C.A., Kemps E., Higgs S., Jones A. (2017). The bogus taste test: Validity as a measure of laboratory food intake. Appetite.

[23] Cappelleri J.C., Bushmakin A.G., Gerber R.A., Leidy N.K., Sexton C.C., Lowe M.R., Karlsson J. (2009). Psychometric analysis of the Three-Factor Eating Questionnaire-R21: Results from a large diverse sample of obese and non-obese participants. Int. J. Obes..

[24] Francis L.J., Brown L.B., Philipchalk R. (1992). The development of an abbreviated form of the revised Eysenck personality questionnaire (EPQR-A): Its use among students in England, Canada, the U.S.A. and Australia. Pers. Individ. Dif..

[25] (2016). IBM SPSS Statistics for Windows.

[26] Kaiser H.F. (1960). The Application of Electronic Computers to Factor Analysis. Educ. Psychol. Meas..

[27] Cattell R.B. (1966). The Scree Test For The Number Of Factors. Multivariate Behav. Res..

[28] Robinson E., Kersbergen I., Higgs S. (2014). Eating “attentively” reduces later energy consumption in overweight and obese females. Br. J. Nutr..

