# Supplementary material for: Remembered Meal Satisfaction, Satiety, and Later Snack Food Intake: A Laboratory Study
Source: Nutrients. 2018 Dec 3;10(12):1883. doi: 10.3390/nu10121883 (PMC6316284; doi:10.3390/nu10121883)
Supplement: Supplementary file 1 [file nutrients-10-01883-s001.pdf]

Supplementary materials for '**Remembered Meal Satisfaction, Satiety and Later Snack Food Intake: A Laboratory Study.'**

**Table S1.** Correlation matrix for memory for satisfaction with the lunchtime meal questions.

| | 1) Overall, how satisfying did you find the lunchtime meal? | 2) Overall, how dissatisfying did you find the lunchtime meal? | 3) How satisfied were you with how filling the lunchtime meal was? | 4) How dissatisfied were you with how filling the lunchtime meal was? | 5) How satisfied were you with the taste of the lunchtime meal? | 6) How dissatisfied were you with the taste of the lunchtime meal? | 7) I liked the lunchtime meal |
|---|---|---|---|---|---|---|---|
| 1 | | -.87** | .30** | -.30** | .84** | -.76** | .83** |
| 2 | | | -.25** | .37** | -.76** | .81** | -.76** |
| 3 | | | | -.84** | .21* | -.18* | .31** |
| 4 | | | | | -.16 | .22* | -.29** |
| 5 | | | | | | -.87** | .81** |
| 6 | | | | | | | -.75** |

* indicates $p < .05$, ** indicates $p < .01$

**Sensitivity analyses**

Excluding those who expected satisfaction with the meal to influence snack intake did not alter the effect of condition on snack intake, $F(2,122) = 0.85$, p = 0.43, $\eta^2 = 0.01$. Including BMI as a covariate did not affect the result for snack intake, $F(1,124) = 0.66$, $p = 0.52$, $\eta^2 = 0.01$, neither did including cognitive restraint, $F(2,124) = 0.50$, $p = 0.61$, $\eta^2 = 0.01$, or uncontrolled eating, $F(2,124) = 0.53$, $p = 0.59$, $\eta^2 = 0.01$.

**Exploratory analyses**

After excluding participants who did not complete the rehearsal task as fully intended (n = 16 satisfying rehearsal condition, n = 12 dissatisfying rehearsal condition) the effect of the rehearsal condition on ad libitum snack food intake remained non-significant, $F(2,97) = .43$, $p = 0.65$, $\eta^2 = 0.01$. See Table S2.

**Table S2**. Snack food intake as a function of recoded rehearsal condition.

| | Neutral rehearsal<br>mean (*SD*)<br>n = 44 | Satisfying rehearsal<br>mean (*SD*)<br>n = 27 | Dissatisfying rehearsal<br>mean (*SD*)<br>n = 29 |
|---|---|---|---|
| Snack food intake (kcal) | 325.49 (156.57) | 302.66 (148.00) | 342.19 (176.46) |
